# Supplementary material for: Are carbon emissions trading and green financial instruments synergistic? -Comprehensive quantitative research based on content analysis
Source: PLoS One. 2024 Mar 7;19(3):e0298601. doi: 10.1371/journal.pone.0298601 (PMC10919601; doi:10.1371/journal.pone.0298601)
Supplement: S1 Data — (ZIP) [file pone.0298601.s001.zip › Data/地级市Z_F工作报告.zip!/╕ú╜¿/─╧╞╜╩╨/└┤╫╘╣1⁄2╓┌║┼í╛┬φ┐╦╩2╛▌═°í┐_2018.docx]

南平市人民政府市长 许维泽

各位代表：

现在，我代表南平市人民政府，向大会报告政府工作，请予审议，并请市政协各位委员和其他列席人员提出意见。

一、2017年主要工作回顾

过去一年，在以习近平同志为核心的党中央坚强领导下，在习近平新时代中国特色社会主义思想的指导下，我市各级政府深入学习贯彻党的十八届三中、四中、五中、六中、七中全会和党的十九大精神，认真贯彻落实省委、省政府和市委的决策部署，坚持稳中求进工作总基调，着力推进供给侧结构性改革，以科学的态度、磅礴的气势开展重大项目、重点工作“百日攻坚战”“四比六促”和“工作落实月”活动，加快建设全国绿色发展示范区，全市经济社会保持平稳健康发展。

初步统计，2017年全市生产总值1632.5亿元、增长7.6%；固定资产投资1985.84亿元、增长17.2%；一般公共预算总收入129.84亿元、增长10.35%，地方一般公共预算收入87.08亿元、增长9.49%；外贸出口95亿元、增长20%，实际利用外资2.34亿美元、增长44.3%；社会消费品零售总额615亿元、增长10.5%；城镇居民、农村居民人均可支配收入30182元、14530元，分别增长8.5%、9%；旅游接待总人数4130万人次、增长20%，旅游总收入602亿元、增长30%。基本完成了市五届人大一次会议确定的目标任务，实现新一届政府良好开局。

（一）绿色发展取得新成效。坚持以新发展理念引领经济发展新常态，牢固树立和践行“绿水青山就是金山银山”的理念，持之以恒走绿色发展道路，发展速度、质量和效益同步提升。

发展思路更加清晰。立足我市资源禀赋和生态优势，邀请国家科技部、中国工程院专家团队，认真梳理出现代绿色农业、旅游、健康养生、生物、数字信息、先进制造、文化创意等七大产业，高水准编制绿色产业发展行动纲要和七大产业规划，出台扶持政策，构建起绿色产业发展体系的“四梁八柱”。启动实施42个、总投资397.55亿元的绿色产业先行项目，发挥示范带动效应。

发展方式加快转变。持续推进产业转型升级，产业结构进一步优化、优势进一步凸显、质量进一步提升。现代绿色农业提质，加快发展品牌农业、智慧农业、生态农业和设施农业，申报“三品一标”农产品39个，注册地理标志商标10件；加快实施“天空地”农业大数据项目，建成农产品质量安全检验检测中心；建设改造高标准农田35.9万亩，建成省级以上农业标准化示范区6个，制定产业标准体系5个；新增省级农民专业合作社32个、省级以上农业产业化龙头企业94家，与中央电视台《食尚大转盘》栏目合作宣传推介我市特色农产品，金砖厦门会晤选用的我市农副产品、伴手礼达52种，成功获得第十四届中国茶业经济年会承办权，获批国家农业可持续发展试验示范区和武夷山国家级田园综合体试点。新型工业提效，坚持创新引领，新增省级“小巨人”企业23家、知识产权优势企业5家，加快实施126个工业投资技改项目、35个智能制造和85个两化融合项目；开展“百名局长帮扶百家企业”活动，精准服务实体经济，新增产值超亿元企业75家；实施品牌和质量标准战略，认定中国驰名商标2件，绿康生化在中小板上市；有效调动金融资源支持企业发展，推广无间贷、流水贷、税贷通等创新信贷产品和服务模式，努力解决企业融资难题；南平高新技术产业园区获省政府审批，市本级三大产业组团、各县（市、区）产业园区基础设施和公共服务设施不断完善，园区产业发展承载能力进一步提升。现代服务业提档，加快发展旅游产业，与兄弟地区组建大武夷旅游联盟，建成邵武花千谷庄园、武夷山马场洲湿地公园、顺昌洋口红色旅游小镇等一批旅游精品，新增2家4A级、15家3A级旅游景区，新建和改建旅游厕所120座，开展5场旅游专项推介，大武夷旅游品牌进一步打响；大力发展健康养生产业，武夷山荣昌汇、国际健康小镇等一批养生项目加快推进；加快发展文化创意产业，建瓯武夷根艺城获批省重点文化产业园区，印象大红袍在“新三板”挂牌；积极推进商贸物流产业，武夷新区城市商贸综合体、光泽冷链物流园等一批商贸物流项目有序推进；持续加快电子商务产业发展，建瓯电商园、南纸电商园等一批电商园区相继建成投入使用。

（二）项目攻坚实现新突破。以开局就是决战、起步就是冲刺的态势，实施重大项目、重点工作“百日攻坚战”，攻坚期间谋划、招商、开工、竣工的项目总数超过1000项，完成征地5.98万亩、拆迁102.19万平方米，解决了项目推进过程中一批长期没有解决的困难和问题。巩固攻坚成果，持续开展“四比六促”“工作落实月”和“千名干部服务千个重点项目”活动。每月集中签约和开竣工一批项目，全市172个在建行动计划重大项目完成投资369.1亿元，382个重点项目完成投资600.9亿元，32个工程包完成投资165亿元；新增列入省级“五个一批”项目3267项，总投资9206亿元，前三季度建阳、建瓯、顺昌、光泽、政和等5个县（市、区）的固定资产投资增幅先后位居全省前十名，南平市在全省“五个一批”项目正向激励综合考评中位居第二名。一大批重大项目提速提效，南平联络线高速公路、太阳电缆建筑用线智能化改造、南纺高新材料产业园、圣农食品深加工等项目建成投产运营，顺邵高速公路、南三龙铁路南平段、衢宁铁路南平段等项目加快推进，武夷山茶旅小镇、延平新城港区、闽江航道整治工程等项目开工建设，武夷山机场迁建、温武吉铁路、武沙高速公路等项目前期有序推进。

持续加大招商引资力度，成功举办绿色发展创新大会以及绿色农业、生物和旅游等产业项目与资本技术对接会、第十一届海峡两岸茶业博览会和首届武夷食品博览会、南平市首届旅游产业发展大会暨旅游产品交易会，积极参加“6·18”项目成果交易会、“9·8”投洽会，赴美国、加拿大以及香港、澳门、深圳、杭州等地开展招商，推行招商专员制度，采取“一把手”招商、产业链招商、第三方招商和以商引商等方式，引进370个总投资超亿元项目。

（三）城乡建设呈现新面貌。以创建省级文明城市为抓手，结合生态修复、城市修补“双修”工作，高效推进武夷新区、延平城区两个增长极和各县（市、区）城区“水美城市”及美丽乡村建设。

按照“满足需要、适度超前、统筹平衡”的要求，围绕行政中心搬迁，建设各类基础、市政和公共服务设施，加快武夷新区开发建设和武夷新区、建阳区同城化发展，集中力量推进南林核心区、将口片区、兴田片区和武夷高新技术园区建设，加快建设轨道交通1号线、303快速通道、绕城高速、水资源配置工程、文化中心、艺术中心、云谷小区一期、武夷文旅广场和市民广场等一批项目，相继建成软件园一期、闽铝轻量化一期和科技孵化器等一批项目；加快建设“十纵十横”交通路网、体育中心等项目，进一步策划梳理出市政道路、景观风貌等方面97个、总投资200亿元的互联互通共享项目，促进武夷新区与建阳区同城融合发展，为启动行政中心搬迁打下坚实基础。

按照“老城做减法、新城做加法、市场运作、自求平衡”的要求，全力推进延平城区建设，完成“三横四纵五片”42条道路“白改黑”31.2公里，以及南平城区至西芹道路改造；完成主要街区建筑立面改造96幢；更换新建公交站点50座、改造28座；改造提升城市公厕69座、新建2座；改造整治昼锦市场等老旧市场4个；改造社区公园19个、新建2个，建成高铁北站站前广场公园；完成文体路、九峰路天桥建设。加快实施滨江路地下通道、九峰和杨真隧道、“三江六岸”景观工程、城市重要节点绿化景观提升以及一批停车场、农超市场项目。开工建设中学岭道路改造工程、316国道快速通道、马头山-九峰山公园等项目，启动实施三中至杨真栈道等一批项目。整治胜利街、鼓楼街等地占道经营、车辆秩序混乱等不文明现象，城市功能和形象得到明显完善和提升。

实施“水美城市”和美丽乡村建设。在全国率先提出“水美城市”建设理念，编制专项规划，采取“PPP+EPC”建设模式，有力推动各县（市、区）城区12个“水美城市”项目建设，完成投资59亿元。国家水利部以我市“水美城市”为标准，制定《全国“水美城市”建设规划导则》。加快建设3个国家级特色小镇，实施美丽乡村建设项目905个，完成千村整治村庄133个、百村示范村庄11个。邵武和平、政和石圳获“中国人居环境范例奖”。

（四）民生福祉得到新改善。基本完成24项为民办实事项目。优先发展教育，实施五大提升工程。

入选省中小学学科教学带头人培养计划56人、省名师名校长培养计划13人，建立健全学生综合素质评价体系，加强学生科学文化素质培养，成立市教育科学研究院，加快建设三级教研网络，新建公办幼儿园13所，创建省级义务教育管理标准化学校59所，遴选3所高中校申报2017年度省级普通高中课程改革基地建设学校，中职学校100%完成标准化创建。加强医疗卫生工作，推进医药卫生体制改革，建立医疗联合体15个，新审批社会资本医疗机构15个，成立市医保局，完成“三保合一”工作，公立医院医疗费用平均增幅控制在10%以内，家庭医生签约服务覆盖率超过30%。加强乡村卫生服务，完成设置一体化管理村卫生所1464所。实施卫技人才储备，招录医学院校定向委培生225名。完善社会保障服务，实施全民参保计划，全市城镇职工基本养老、失业、工伤和职工居民医疗保险参保分别达到65.1万人、37.15万人、53.5万人、294.13万人。新增城镇就业2.06万人，城镇登记失业率控制在2.99%以内，劳动保障监察“两网化”管理实现全覆盖。基本建成各类保障性住房4261套，配租配售1983套。落实社会福利、慈善事业等制度，健全完善妇女儿童、老年人和残疾人关爱服务体系。推动文体事业发展，举办“五个一百”“朱子四礼”和建窑建盏文化博览会等活动，加快推进“朱子文化大观园”和四所朱子书院等项目建设，进一步打响特色文化品牌。群众体育和竞技体育协调发展，第四届武夷山国际马拉松等赛事成功举办。深化“平安南平”建设，刑事发案大幅下降，生产安全事故总量持续下降，全市人民群众安全感率达95.17%。重视食品安全，启动“国家食品安全示范城市”创建申报工作。有效处置不良贷款，辖区不良贷款率控制在2.99%以内。同时，民族宗教、外事侨务、港澳台合作、气象防震、人民防空、档案方志、老区库区等工作继续加强。军政军民关系融洽和谐，连续七届被评为省级双拥模范城。

（五）生态环境厚植新优势。扎实推进国家生态文明试验区建设各项试点任务，武夷山国家公园体制工作取得新进展，乡镇党政领导干部自然资源资产离任审计工作在全省推广。加快建设总投资215.3亿元的国家储备林质量精准提升工程，成功申报国家山水林田湖草生态保护修复试点，超额完成年度能源消耗总量和强度“双控”任务，顺利通过国家财政部节能减排综合示范三年总评。强力实施水污染防治行动计划，全面落实“河长制”，全市共设区域河长150名、流域河长525名；按照“立即关、坚决拆、综合治、长效管”的要求，开展畜禽养殖污染专项整治行动，全市共拆除生猪养殖场7180家，削减生猪293.86万头；着手采取PPP工程包的模式启动推进城乡污水、垃圾治理，主要水系水质状况为优；严厉打击河道非法采砂，取缔非法采砂点185个；全面开展排污权有偿使用和交易工作，实现排污权交易出让8405万元。深入实施大气污染防治行动计划，提前超额完成黄标车淘汰任务，淘汰燃煤锅炉426台，荣华山产业组团合成革企业“煤改气”和DMF回收取得阶段性成效，顺昌、邵武、建阳、政和以及洋口国有林场纳入全省林业碳汇交易试点。积极实施土壤污染防治行动计划，治理水土流失39.59万亩，加快推进建阳和邵武土壤污染治理与修复试点示范工程；严厉打击非法违法采矿，关闭取缔非法采矿点3个，完成矿山治理面积41.48公顷。全面实施高铁、高速公路及江河沿线的环境整治和景观提升工程，生态环境明显改善。

（六）精准扶贫进入新阶段。强化脱贫责任，深化挂钩帮扶机制，逐户落实精准脱贫措施。加大产业扶贫、就业扶贫力度，“百企帮百村”活动扎实开展，“雨露计划”“春潮行动”共培训贫困人口1.6万人次。建立精准医疗扶贫慈善救助、精准扶贫医疗叠加保险等制度, 8.97万农村贫困人口得到医疗补助。加强兜底保障，农村低保标准由家庭年人均收入2650元提高到3750元。加快造福工程易地扶贫搬迁进程，建设集中安置区41个，完成建房人数达1.34万人。完成贫困村的旧村复垦项目548.18亩，加快贫困村水利设施、路网工程建设，贫困村生产生活条件明显改善。全年脱贫3.77万人，光泽县以及全市178个贫困村实现脱贫摘帽。

（七）改革创新释放新活力。加快推进城市执法体制、旅游管理体制、安全生产领域以及供销社改革，全面启动环保机构监测执法垂直管理制度改革。深化“放管服”改革，前置审批事项精简18%，中介服务事项精简42%；全面实施“双随机一公开”监管，实现权责清单市、县、乡三级政府全覆盖；在全省率先开展“一窗受理、集成服务”工作，建设“五级一体”放管服“云平台”，93.46%的审批服务事项实现“最多跑一趟”和“一趟不用跑”。着手设立产业发展基金、股权投资基金、PPP引导基金，从资金上支持不同发展阶段的企业。分类推进市属国有企业改革，注入有效资产提高国有企业信用等级，实施经营性项目、非经营性项目分类考核，促进国有企业做强做优做大。制定人才工作十条措施和六份配套政策文件，出台激发本土人才干事创业活力二十条措施，开展“人才·南平校园行”专场招聘等活动，破解人才紧缺瓶颈。稳妥实施农村土地“三权分置”，加快推进土地确权颁证，1645个村完成权属调查。继续推行“三级联动、重心下移”联系服务群众工作机制，创新完善新时代科技特派员制度，全国深入推行科技特派员现场会在我市召开。

（八）自身建设开创新局面。各级政府深入开展“两学一做”学习教育活动，结合学习全国优秀共产党员、时代楷模廖俊波精神，广泛开展“一月一主题”学习讨论活动。认真落实中央八项规定精神，有效控制“三公”经费，全面实施党政机关公务用车制度改革。进一步深化效能建设，建立全市重点工作责任清单和问题清单督查平台，全面推动工作提速和服务提质。主动接受人大监督、政协监督和社会监督，坚持向人大及其常委会报告工作，向政协通报工作情况，办理人大代表议案1件、建议222件，政协委员提案294件，满意和基本满意率均为100%。提请市人大审议通过《南平市朱子文化遗存保护条例》《南平市市容和环境卫生管理办法》。认真吸纳市政协开展的加强村庄小溪流治理、推进“回归”工程、发展红色旅游、净化校园周边环境等4个重点协商意见。坚持依法治市，深入推进依法行政，加大行政监察、审计监督和政务公开力度，扎实推进党风廉政建设和反腐败工作。

各位代表，过去一年的成绩来之不易。这是省委、省政府和市委正确领导的结果，是全市人民齐心协力、奋力拼搏的结果。在此，我代表市人民政府，向全市人民，向各民主党派、各人民团体和社会各界人士，向中央和国家机关以及省直机关驻地机构，向人民解放军和武警驻地部队，向关心支持南平发展的港澳同胞、台湾同胞、海外侨胞和国际友人，致以崇高的敬意和表示衷心的感谢！

在肯定成绩的同时，我们也要清醒地认识到发展中还存在着不少困难和问题。主要是：工业经济总量偏小，工业转型升级步伐不快、发展的质量和效益不高，现代服务业发展水平较低、消费需求不旺；大项目好项目接续不足，自主创新能力不够，新的经济增长点支撑能力不强，有效投资结构亟需优化；城市化水平低，对资本、科技、人才等要素吸附能力弱，城市规范化、精细化管理还存在不少问题；民生领域欠账较多，教育、医疗等方面的短板仍然存在，优质公共服务供给不足；一些干部缺乏敢于担当、勇于拼闯、积极作为和主动服务的意识。对以上问题，我们将尽最大努力认真加以解决。

二、2018年发展目标任务

党的十九大开启了新时代中国特色社会主义事业的新征程，习近平总书记作为党中央的核心、全党的核心，是全党同志的共同意志，全国各族人民的共同意愿。我们要牢固树立“四个意识”，坚定“四个自信”，让习近平新时代中国特色社会主义思想在南平落地生根、开花结果。

2018年是贯彻党的十九大精神的开局之年，是改革开放40周年，是决胜全面建成小康社会、实施“十三五”规划承上启下的关键一年。政府工作的总体要求是：全面贯彻党的十九大精神，以习近平新时代中国特色社会主义思想为指导，坚持稳中求进工作总基调，坚持新发展理念，按照高质量发展的要求，统筹推进“五位一体”总体布局和协调推进“四个全面”战略布局，坚持以供给侧结构性改革为主线，统筹推进稳增长、促改革、调结构、惠民生、防风险等各项工作，打好防范化解重大风险、精准脱贫、污染防治攻坚战，全力推进南平高质量发展、赶超发展、绿色发展。

2018年经济社会发展的主要预期目标是：全市生产总值增长8.5%，固定资产投资增长16%，一般公共预算总收入增长10.14%，地方一般公共预算收入增长10.25%，外贸出口增长5%，实际利用外资总量与上年持平，社会消费品零售总额增长10.6%，城镇居民人均可支配收入增长8.8%，农村居民人均可支配收入增长9.3%。

围绕上述目标，我们要奋力抓好以下八个方面的工作：

（一）加快发展绿色产业，提升经济发展质量

绿色和生态是南平最大的特色和优势。我们要立足特色、发挥优势，进一步优化产业结构，提升创新能力，构建“绿色+科技+互联网”的现代化产业体系。

壮大绿色产业。按照绿色产业发展行动纲要、七大产业发展规划要求，加快实施294个、总投资2033亿元的绿色产业发展支撑项目，促进七大产业互促共融、加快发展。现代绿色农业，按照“质量兴农、绿色兴农”要求，加快国家农业可持续发展试验示范区、武夷山国家级田园综合体试点工作，促进一、二、三产融合发展。加快建设一批粮食产能项目，强化粮食安全生产保障。大力发展茶叶、果蔬、笋制品、食用菌、中草药材等特色种养业，用智能化、信息化手段推进农副产品精深加工，积极培育农村电子商务、休闲观光农业等新型业态，重点推进“天空地”农业大数据、圣农第二祖代种鸡场等项目建设。旅游产业，进一步优化大环境，加强景区景观风貌整治，提升景点通达性，推动旅游服务规范化、标准化；进一步打造大产品，大力发展文化研学旅游、休闲养生旅游、体育健康旅游和民俗乡村旅游，着力引进建设一批大型旅游综合体，重点推进武夷山茶旅小镇、下梅旅游综合体、延平湖水上游等项目建设，加快打造国际养生休闲度假城市；进一步实施大营销，发挥武夷山“双世遗”和国家公园品牌优势，开展借智营销、媒体营销、IP营销，提升大武夷旅游影响力和吸引力。健康养生产业，重点发展生态休闲养生、体育养生、中医药养生、文化修心养生，大力引进国内外高水平医疗健康养生机构，重点推进武夷山国际健康小镇、建阳武夷德懋堂、荣华山医养健康园等项目建设。生物产业，优先发展生物农业、生物制造、生物医药和生物能源产业，重点推进圣维兽药疫苗、广生堂乙肝原料药、承天中药材种植与加工产业园等项目建设。数字信息产业，突出培育特色软件和信息服务业，探索发展云计算、大数据、物联网等新兴产业，重点推进智慧南平时空大数据与云平台、邵武物联网处理中心等项目建设。先进制造业，加快推动新型轻纺、新型建材、精细化工、竹木加工等传统制造业转型升级，促进汽车汽配、电力装备、食品加工装备等三大先进装备制造业发展壮大，拓展新材料、新能源、智能制造装备等新兴产业，重点推进邵武永晶氟系列高新材料、建瓯利树白木浆挂面纸、松溪闽瑞新型复合纤维等项目建设。文化创意产业，加快发展文化会展、娱乐演艺、影视传媒、工艺美术、工业设计等产业，重点推进五夫朱子文化园、考亭大宋文化生态书苑、邵武樱花文化特色小镇等项目建设。

振兴实体经济。统筹推进科技链、产业链、金融链和政策链“四链融合”，支持企业做大做强，带动产业发展。坚持创新驱动，集中资源打造一批“双高”龙头、新技术和科技小巨人企业群体，力争新认定国家级企业技术中心1个，省级企业技术中心和工业设计中心各2个，高新技术企业50家，科技型企业250家，创新型企业26家。开展质量提升行动，支持企业争创中国驰名商标、国家地理标志商标、全国和省级质量奖、省著名商标及名牌产品，鼓励企业参与各领域国家标准、行业标准的制修订工作，提升行业话语权。持续完善产业平台，按照“生产专业化、生活社区化、环境生态化”的要求，加快推进武夷高新技术园区、南平工业园区、荣华山产业组团及各县（市、区）园区平台的生产生活配套建设，提升服务水平和投资效益。引导金融支持实体经济，完善政银企联席会议制度，鼓励金融机构创新金融产品，发挥各类基金作用，支持企业直接上市融资，拓宽中小企业融资渠道。

与此同时，积极营造企业家健康成长环境，弘扬优秀企业家精神，培育一批具有诚信品质、创新意识和敢于拼搏的企业家队伍。大力支持本土企业发展，增强内生发展动力，本土企业根植南平，与南平血脉相连，我们更要高看一眼、厚爱一层，让他们在南平这片沃土上茁壮成长、根深叶茂。

实施精准招商。围绕七大产业，瞄准国际国内500强、行业龙头、知名企业，继续落实招商专员制度，强化“一把手”招商、龙头招商、平台招商、以商招商，全年签约超亿元合同项目300个、新开工超2000万元项目300个以上。坚持引资引智并举，运用各类重大展会和对接平台，吸引一批行业领军人物、学科带头人，带着先进理念、技术团队和高新技术项目落户南平。发挥香港南平联谊总会、澳门南平联谊会以及异地商会作用，深入实施“回归工程”，引进一批人才、资金和项目反哺支持家乡建设。创新对台合作模式，在产业、文化、科技等方面进行深入对接。主动融入“一带一路”战略，积极参与闽东北经济协作区、闽浙赣皖福州经济协作区和闽浙赣皖九方经济协作区经贸交流合作。

（二）加快建设水美城市，打造宜居宜业家园

城市让生活更美好，把城市建设得更漂亮、更宜居、更有归属感，是全市人民的期盼。要全面加强城市规划建设管理，统筹加快武夷新区、延平城区和县域中心城市建设，打造城在水中、景在城中、山水相融、城景相依的“水美城市”。要持续推进“厕所革命”，努力补齐影响群众生活品质的短板，不断改善群众生产生活环境，巩固省级文明城市创建成果。

全面加快武夷新区建设。按照“市区一体、规划统一、建设多元、管理下沉”的原则，全力推进武夷新区开发建设以及武夷新区和建阳区同城化发展。南林核心区要围绕打造集行政、文化、商业、居住于一体的新兴城市功能区，加快建设“一纵三横”路网、武夷文旅广场、政务服务中心、云谷小区二期等一批重点项目。将口片区要重点发展文化休闲、体育康养、物流商贸等产业，结合崇阳溪景观带建设，加快高铁站前广场片区开发建设，尽快推动马拉松小镇等一批项目落地，打造集体育、文化、旅游为一体的崇阳溪特色产业走廊。兴田片区要重点发展文化旅游、度假养生和会展创意等产业，全力推进森林生态博物院等项目建设。武夷高新技术园区作为城市副中心，要加快完善公共服务配套，全力打造武夷智谷特色小镇，积极推进软件园二期、闽铝轻量化二期等项目建成投产，加快推动建窑建盏展示交易中心、海源新材料三期、闽北物流快递分拨中心等一批项目开工建设。推动同城化发展要合理布局建设基础设施、市政设施和公共服务设施，加快建设“十纵十横”交通路网、公交总站、体育中心、轨道交通2号线等项目，促进武夷新区与建阳区互联互通、共建共享。2018年春，科学有序启动行政中心搬迁。

持续推进延平城区建设。坚持“老城补短板、新城强功能、新老城互通、产城共融合”的原则，全力促进延平城区做精做美。老城区继续实施道路“白改黑”、立面改造、厕所改造和老旧小区整治提升工程，加快建设一批道路、桥梁、隧道、停车场、农超市场、城市公园等项目，加大城区占道经营、车辆无序停放、违章搭盖等市容环境整治管理力度，补齐城市短板。新城区大力推进小鸠片区、高铁南平北站片区综合开发，加快延平新城港区、斜溪湿地公园建设。围绕新老城区互通，加快建设316国道快速通道、“三江六岸”景观提升、夜景夜游工程、马头山-九峰山公园等项目，开工建设滨江东路。加快建设南平工业园区，引进高新技术产业和一批“五南”企业的中下游产业项目，配套建设教育、医疗、文化等设施，完善城市功能，促进产城融合。

大力推进县域中心城区建设。按照“城市提升、产业发展、生态保护、水利防洪相融合”的原则，继续高起点规划、高标准设计、高质量建设10个县（市、区）的“水美城市”。突出河畅、水清、岸绿，注重水面景观与岸上景观、白天景观与夜间景观、历史文化景观与现代文明景观相结合。树立经营城市的理念，着力实施造水、造景、造城、造业、造才等“五造”工程，带动“山、水、城、人”一体发展，为全国推广“水美城市”建设制定标准、树立标杆。

（三）加快实施项目带动，持续扩大有效投资

项目是经济社会发展的载体，要积极开展“项目突破年”活动，以更大的力度、更高的效率、更实的举措谋划推进一批大项目、好项目。全年新增“五个一批”项目1500个；实施省市重点项目428个，年度计划投资634.6亿元。

促进项目滚动接续。对标经济发达地区，全面梳理基础设施、产业发展、民生社会事业等发展重点和薄弱环节，采取“一把手”亲自策划、引导企业策划、委托专业咨询机构策划等方式，全方位、多层次谋划一批具有前瞻性、拉动力强的重大项目，努力对接引进、开工投产一批大项目。力争温武吉铁路、宁德至南平铁路、武夷山机场迁建、上饶至浦城高速公路等一批重大项目前期工作取得明显进展，开工建设武沙高速公路、邵武永椿新材料等项目，加快建设衢宁铁路、南平生活垃圾焚烧发电厂、元力高端精制活性炭等项目，推动南三龙铁路、顺邵高速公路、荣华山现代物流园、海源汽车零部件等项目竣工和投产。

健全项目推进机制。加强“五个一批”项目策划管理，完善项目策划联席会议和评审机制、领导挂点重大项目、重点前期项目“四个一”和“五个一批”项目正向激励等机制。持续开展“千名干部服务千个重点项目”活动，实行“一月一协调、一季一督查”，加大对项目的考核评比力度，继续开展每月一次的项目集中签约和集中开竣工活动，每月公布项目进展情况，千方百计推动项目落地建设、投产达效。

强化项目要素保障。加大前期投入，通过项目前期购买服务、集中会审、EPC建设等方式，加快策划项目的转化升级。建立重大项目审批服务“绿色通道”，积极推行并联审批、交叉审批，促进项目审批提速提效。通过发行债券、融资租赁、PPP等方式，拓宽项目融资渠道。以会战的方式强力推进征地拆迁，积极向上争取项目用地、用林及环境容量等要素指标，保障项目建设需求。

（四）加快推进乡村振兴，推动农业农村发展

按照“产业兴旺、生态宜居、乡风文明、治理有效、生活富裕”的要求，坚持农业农村优先发展，让农业成为有奔头的产业，让农民成为有吸引力的职业，让农村成为安居乐业的美丽家园。

发展乡村经济。构建现代农业产业体系、生产体系、经营体系，完善农业支持保护制度，大力培育龙头企业、专业合作社、家庭农场和种养大户等新型农业经营主体，完善利益联结机制，带动农户“抱团”发展。盘活农村集体资产，提高各类资源要素的配置和利用效率，多途径、多渠道发展壮大集体经济。巩固和完善农村基本经营制度，推进农村土地制度改革，完善农村承包地“三权分置”制度。推行土地入股、土地流转、土地托管等多种经营方式，大力发展市场化、专业化、多元化的农业生产性服务业，保持乡村经济发展的旺盛活力。

改善乡村环境。在深入开展“万人保洁”的基础上，推行市场化乡村污水垃圾治理，整治农村违法占地、违规建房，让乡村更干净、更整洁。加强农村“四好”公路建设，全面推行“路长制”。按照“典型示范、以点串线、梯级推进、彰显特色”的思路，改造提升传统民居、老旧院落、农村厕所，保留乡村自然风貌、村庄肌理和乡愁记忆，全面建成65个精品示范村，打造一批美丽庭院、美丽农庄、美丽田园。

完善乡村治理。加强农村基层基础工作，健全自治、法治、德治相结合的乡村治理体系。加强农村自我服务管理，提升村民自觉参与乡村治理的水平，组织好新一轮村级组织换届选举工作。强化农村法治宣传，依法打击农村违法乱纪现象，促进农村公平正义。开展移风易俗活动，全面推进乡风文明。注重培养驻村干部、大学生村官、“三支一扶”、大学生志愿者和基层党群工作者等队伍，推动新时代科技特派员制度创新发展，赋予制度新内涵，拓展服务新领域，造就一支懂农业、爱农村、爱农民的“三农”工作队伍。

（五）加快生态文明建设，全力打造美丽南平

坚持人与自然和谐共生，像对待生命一样对待生态环境，严守生态红线，保护生态环境，努力以生态的“高颜值”推动发展的“高素质”。

加快推进生态文明试验区建设。继续落实国家生态文明试验区建设赋予我市的各项任务，率先设立国有自然资源资产管理机构，全面推行自然资源资产负债表编制、生态环境损害赔偿等制度，完成生态系统价值核算试点，进一步总结推广乡镇领导干部自然资源资产离任审计做法。认真实施国家储备林质量精准提升工程，加快林木栽培、改培和赎买工作，优化林分空间结构，完善森林生态功能，落实国家森林城市创建各项工作。加快实施国家山水林田湖草生态保护修复试点工作，积极发行绿色专项债券，探索建立生态银行，力争形成一批可复制、可推广的经验成果。

加强环境综合整治和生态保护。全面落实“水十条”“气十条”“土十条”和“河长制”，持续实施水、大气、土壤污染防治管控和修复工程。加快编制全市河道岸线及河岸生态保护蓝线规划，保护好沿岸生态环境，加强饮用水源地保护，持续抓好畜禽养殖污染整治、小流域环境综合整治，加快实施城乡污水处理。加大节能减排力度，大力推进企业、园区循环化改造和新能源汽车推广应用。加快矿山生态环境治理恢复，推动固体废弃物和垃圾减量化、资源化、无害化处理，逐步实施垃圾分类处理，扎实做好水土流失治理、湿地保护恢复、地质灾害防治和农村面源污染整治。完成10个县（市、区）能耗“双控”目标，加强监测网络建设，进一步巩固中央、省环保督察成果。

（六）加快实施精准扶贫，如期完成脱贫任务

要坚持党政“一把手”负总责、各级干部层层负责的工作制度，注重扶贫与扶志、扶智相结合，把脱贫工作精准落实到村、到户、到具体贫困人口，确保3个贫困县、96个贫困村脱贫摘帽。

实施产业、金融扶贫，完善龙头企业牵引、合作组织链接、资产经营分享、创业支持服务等机制，创新“政府+企业+村级组织+贫困农户”的产业扶贫模式，鼓励贫困户利用既有的资源、资产参与产业发展，增加贫困户经营性和资产性收入。促进扶贫小额信贷扩面增量，使更多贫困群众获得金融支持。继续开展“雨露计划”“春潮行动”，全年培训2.4万人次，让贫困户掌握生产技能，增强脱贫能力。实施健康、教育扶贫，用好精准扶贫医疗叠加保险、精准医疗扶贫慈善救助等政策，切实降低因病致贫、因病返贫的风险；增强贫困地区师资力量，深化贫困生联系帮扶制度，让贫困户的子女都上得起学，从源头上阻断贫困代际传递。实施社会力量扶贫。整合各类扶贫资金、资源和项目，加快实施贫困村提升工程，持续开展扶持村级集体经济发展试点工作，促进贫困村和谐稳定发展。鼓励支持个人、企业和社会组织参与扶贫开发，开展村企互动助力脱贫和向贫困地区、贫困人口献爱心活动。加大政策性保障兜底力度，逐步提高保障水平，帮助没有收入和没有劳动能力的贫困人口脱贫。同时，继续做好贫困人口的动态管理，及时对遗漏、脱贫不稳和返贫的贫困人口进行再识别，做到应统尽统、应扶尽扶。对刚脱贫的困难群众，要留出缓冲期，“扶上马再送一程”，做到扶贫政策不变、扶贫项目不断、扶贫资金不减、智力帮扶不停，让贫困人口在脱贫致富的道路上行稳致远。

（七）加快发展社会事业，切实保障改善民生

始终把人民群众的小事当作我们的大事来做，围绕群众最关心、最直接、最现实的问题，完善公共服务体系，努力满足群众日益增长的美好生活需要。

打赢教育翻身仗。始终把教育放在优先发展位置，持续开展五大提升工程，着力解决中小学生课外负担重、“择校热”和“大班额”等突出问题，不断提高教育教学质量。加强教师队伍建设，推进教师“县管校聘”改革，全面实施教师资格定期注册制度，继续开展名师、学科带头人培养工程和骨干教师培训计划，提升教师职业水平。发展普惠性学前教育，探索政府购买普惠性学前教育服务，开工建设10所公办幼儿园，新增学位2800个。推动义务教育优质均衡，加快实施小学“强基”、 初中“壮腰”和中小学扩容工程，创建50所义务教育管理标准化学校。实施高中多样化优质化发展计划，引进一批优质资源开展高中教育战略合作，培育2所普通高中课程改革研究基地，加快建设武夷新区高中学校，力争南平一中、建阳一中、建瓯一中创建省级示范性高中。加快职业教育联盟集团化，鼓励和支持武夷学院参与职业教育联盟，不断完善现代职业教育体系。

加快健康南平建设。实施医疗卫生补短板“八大工程”，继续解决好“看病难、看病贵”问题。深化医药卫生体制改革，落实分级诊疗制度，支持市属公立医院组建紧密型医疗集团，推进县域紧密型医联体建设；加强乡村卫生服务一体化管理，健全基层医疗卫生服务体系；实施“43113”全民健康信息化工程，破解医疗信息数据“孤岛化”“碎片化”问题。优化医疗资源配置，加快推进武夷新区综合医院、市第一医院病房综合楼等项目建设，新建10个以上中医馆，实现每千名常住人口医疗机构床位数达6.26张。加强卫技人才队伍建设，定向培养一批本土基层卫技人才，免费规范化培训一批乡村医生。加强公共卫生工作，加大重大疾病预防控制力度，开展爱国卫生运动。继续稳妥实施全面两孩政策，改进计划生育服务管理，促进人口长期均衡发展。

推动文体事业繁荣。践行社会主义核心价值观，深入挖掘南平优秀传统文化蕴含的思想观念、人文精神、道德规范，结合新时代要求继承创新，让南平文化展现出永久魅力和时代风采。加强文化遗产保护与传承，大力弘扬朱子文化、闽越文化、红色文化、茶文化等特色文化，深度开发建盏、建本、根艺、版画、剪纸、竹木工艺等特色文化产品，办好闽浙赣皖四省四市民间艺术节，提升文化品牌竞争力。完善公共文化服务体系，建成博物馆、图书馆、文化艺术馆、大剧院、南词艺术传承发展中心等一批综合性文化服务中心。落实全民健身战略，加快发展群众体育和竞技体育，培育壮大体育产业，完善一批体育场馆，积极申办第十七届省运会，打造武夷山马拉松、自行车骑游等一批富有影响力的品牌体育赛事。

全面加强社会治理。深入实施“七五”普法，推进公共服务法律体系建设，健全立体化社会治安防控体系，进一步深化社会治理机制，持续开展“平安南平”系列专项行动，加快“雪亮工程”和各级综治中心建设，推动社会治理重心向基层下移，完善信访工作依法处理机制。深入推进安全生产领域改革，加强安全生产隐患防控和系统治理，坚决遏制重特大安全事故。严格食品药品安全监管，深化治理“餐桌污染”，全力打造“食品放心工程”。加强应急管理体系建设，努力提升防灾减灾救灾能力。加大金融风险防范力度，采取有效措施处置不良资产，支持银行对不良资产进行打包转让、核销，严厉打击和处置非法集资、逃废债、虚假租赁等违法行为，加强大宗商品交易场所、互联网金融的监管，维护金融运行稳定，坚决守住不发生系统性金融风险的底线。进一步营造和谐稳定的社会环境，提升群众安全感。

完善社会保障体系。健全城镇职工和城乡居民基本养老保险、基本医疗保险和大病保险制度，全面建成覆盖城乡居民的社会保障体系。完善失业、工伤保险制度，健全就业创业服务体系。统筹城乡社会救助体系，完善最低生活保障制度。重视老年和妇女儿童工作，发展老年体育、教育事业，加快建设完善一批老年日间照料中心、“妇女之家”和“儿童之家”，全面完成乡镇敬老院整改提升任务，力争建设200个以上农村幸福院。持续实施保障性安居工程，建设棚改安置房2471套，加快建立多主体供给、多渠道保障、租购并举的住房制度。

（八）加快体制机制改革，有效激活发展动能

改革创新是发展的强大动力和不竭源泉，要深入推进重点领域和关键环节的改革，通过改革激发活力、释放红利。

要深化“放管服+互联网”改革，完善权责清单管理系统、“四库合一”监管平台和“一网通办”审批模式，加快建设放管服“云平台”，让企业不出园区、居民不出小区就能够享受到最优质的行政服务。按照“既要尽力而为，又要量力而行”的原则，重视政府性债务管理，加强对政府性债务风险事前、事中、事后管理和监督，处理好加快发展与合理负债的关系，加快建立规范透明、标准科学、约束有力的预算制度，促进财政健康、可持续发展。改进财政资金投入方式，创新投融资体制，建立各类发展基金，运用公司化、实体化运作方式，服务实体经济发展和基础设施建设。

继续深化国有企业改革，提升国有企业核心竞争力，推动发展混合所有制经济，促进国有资产保值增值、做强做优做大，防止国有资产流失。推行市场准入负面清单制度，大力支持民营企业发展。继续推进落实商事制度、环保监测执法、审计和统计体制等制度改革。理顺市本级与延平区和建阳区规划、土地、财政和城市管理等体制机制，提升市、区两级服务管理水平，推动市、区一体化发展。

与此同时，继续深化与各民主党派、工商联和无党派人士的合作共事和民主协商，支持工会、共青团、妇联等群团组织发挥更大作用，推动社会组织多元健康发展。持续做好民族宗教、外事侨务、港澳台合作、档案方志、气象防震、老区库区等各项工作。支持驻军部队和武警部队建设，强化国防教育、国防动员、人民防空、反走私、双拥优抚安置等工作，促进军民融合发展。

三、努力打造人民满意的服务型政府

各级政府要深刻领会、深入贯彻习近平新时代中国特色社会主义思想，切实用党的创新理论武装头脑、指导实践、推动工作。要坚持党对一切工作的领导，把党的领导落实到推动经济、政治、文化、社会、生态建设的各项工作中。要始终牢记宗旨，公正用权、改进作风、廉洁从政，切实做到对人民负责、受人民监督、让人民满意。

（一）努力建设法治政府。坚持依法行政，推进行政决策科学化、民主化、法治化，做到法定职责必须为、法无授权不可为，构建依法行政制度体系。深化政务公开，促进行政权力在阳光下运行，以政府诚信带动社会诚信。依法接受人大及其常委会的监督，自觉接受政协的民主监督，主动接受司法、舆论、社会监督。广泛听取各民主党派、工商联、无党派人士、各人民团体和老干部的意见建议，提高人大代表议案建议和政协提案的办理质量。

（二）努力打造高效政府。大力弘扬“马上就办、真抓实干”的作风，实行工作目标管理责任制，强化效能督查，完善绩效评估，充分运用正向激励、目标倒逼、容错纠错和问效问责等机制，鼓励广大干部用心尽责、敢于担当，形成干事创业的良好氛围，确保中央和省、市各项决策部署有效落实。加强干部教育培训，学习借鉴先进地区的好经验好做法，学习掌握经济管理、社会治理和政策法规知识，增强干部队伍推动发展的能力。

（三）努力铸就廉洁政府。按照习近平总书记关于进一步纠正“四风”、加强作风建设重要批示精神，进一步落实中央八项规定精神，驰而不息抓好“四风”整治。持续推进“两学一做”学习教育常态化制度化，全面开展“不忘初心、牢记使命”主题教育，深入学习全国优秀共产党员、时代楷模廖俊波精神，凝聚向上向善的正能量。继续坚定不移地落实全面从严治党，严格履行“一岗双责”，深入推动党风廉政建设和反腐败工作，树立风清气正的政府形象。

各位代表！迈入新时代，踏上新征程，让我们更加紧密地团结在以习近平同志为核心的党中央周围，在省委、省政府和市委的坚强领导下，团结依靠全市人民，不忘初心，砥砺前行，为“再上新台阶、建设新南平”而努力奋斗！
